# Sled-Pull Load–Velocity Profiling and Implications for Sprint Training Prescription in Young Male Athletes

**DOI:** 10.3390/sports7050119

**Published:** 2019-05-20

**Authors:** Micheál J. Cahill, Jon L. Oliver, John B. Cronin, Kenneth P. Clark, Matt R. Cross, Rhodri S. Lloyd

**Affiliations:** 1 Athlete Training and Health, Plano, TX 75024, USA; 2 Sports Performance Research Institute New Zealand, Auckland University of Technology, 0632 Auckland, New Zealand; joliver@cardiffmet.ac.uk (J.L.O.); john.cronin@aut.ac.nz (J.B.C.); cross.matt.r@gmail.com (M.R.C.); rlloyd@cardiffmet.ac.uk (R.S.L.); 3 Cardiff School of Sport, Cardiff Metropolitan University, Wales CF23 6XD, UK; 4 Department of Kinesiology, West Chester University, West Chester, PA 19383, USA; kclark@wcupa.edu; 5 Laboratoire Interuniversitaire de Biologie de la Motricité, University Savoie Mont Blanc, 73000 Chambéry, France; 6 Center for Sport Science and Human Performance, Waikato Institute of Technology, 3200 Hamilton, New Zealand

**Keywords:** resisted sled sprinting, acceleration, horizontal strength training, reliability

## Abstract

The purpose of this study was to examine the usefulness of individual load–velocity profiles and the between-athlete variation using the decrement in maximal velocity (Vdec) approach to prescribe training loads in resisted sled pulling in young athletes. Seventy high school, team sport, male athletes (age 16.7 ± 0.8 years) were recruited for the study. All participants performed one un-resisted and four resisted sled-pull sprints with incremental resistance of 20% BM. Maximal velocity was measured with a radar gun during each sprint and the load–velocity relationship established for each participant. A subset of 15 participants was used to examine the reliability of sled pulling on three separate occasions. For all individual participants, the load–velocity relationship was highly linear (*r* > 0.95). The slope of the load–velocity relationship was found to be reliable (coefficient of variation (CV) = 3.1%), with the loads that caused a decrement in velocity of 10, 25, 50, and 75% also found to be reliable (CVs = <5%). However, there was a large between-participant variation (95% confidence intervals (CIs)) in the load that caused a given Vdec, with loads of 14–21% body mass (% BM) causing a Vdec of 10%, 36–53% BM causing a Vdec of 25%, 71–107% BM causing a Vdec of 50%, and 107–160% BM causing a Vdec of 75%. The Vdec method can be reliably used to prescribe sled-pulling loads in young athletes, but practitioners should be aware that the load required to cause a given Vdec is highly individualized.

## 1. Introduction

The majority of sprint training research has examined the utility of resistance training and plyometrics as methods to enhance sprinting capability [1,2,3] rather than sprint-specific training. Sprint-specific training can be defined as training that is specific to the movement patterns and direction of sprinting and it is likely to be more successful than non-specific training in improving speed [4]. A popular method of sprint-specific training is to add resistance while moving in a horizontal plane of motion, commonly referred to as resisted sprint. Recently, researchers have focused on resisted sled sprinting, specifically sled pulling, as a popular and effective method of sprint training [5,6]. As with traditional resistance training, the resistive load used during sled pulling needs to be appropriately prescribed to cause the desired training adaptations. The majority of previous sled pulling research has been studied in adult populations and has prescribed lighter loads (<30% body mass) with the emphasis on ensuring minimal disruption in sprint mechanics and small acute reductions in speed [7,8]. The reliability of resisted sled sprinting has been studied in adult populations [5]. However, the reliability across multiple loads from light to heavy has not been examined in young athletes. More recently, researchers have used heavier loads (>30% body mass) with the intention of improving horizontal force application [9,10,11]. A review of available research in adults demonstrated that heavier loads have been shown to provide greater increase in initial acceleration when compared to lighter loads during resisted sled pulling [12]. However, there is a paucity of research at loads greater than 20 percent body mass (% BM) in young athletes [13]. Thus, limited insights and practical applications for coaches regarding the effects of sled-pull loading are available for young athletes.

Traditionally, the load applied during sled pulling has been prescribed as a % BM [12]. However, due to differences in size, sex, strength, and training history across athletes, this may be inappropriate [13]. The effects on growth and maturation during adolescence can lead to increased variability in response to resisted sprinting [14]. This is particularly the case in athletes where loading by a given % BM has been shown to slow immature boys by 50% more than mature boys [15]. Consequently, prescribing resistance solely as a % BM is likely to provide an even greater varied training stimulus across young athletes in comparison to adults, providing a limited approach which may lead to adaptations which are not necessarily intended. Given the linear relationship between load and decrement in maximal velocity (Vdec), the Vdec approach has been suggested as a more appropriate way to prescribe resistive sprint loads in comparison to % BM [16]. This method has been assessed through multiple and single sprint trial methods of sled load prescription with both methods proving to be effective in calculating the load that optimizes power (Lopt) during sled pulling [9,16]. As per recommendation by Cross et al. [9], a practical application for coaches is to use a combination of both multiple-trial and single-trial methods. Athletes are assessed performing one single maximum sprint and multiple sled sprints across a range of loads, with data then used to establish individual load–velocity profiles. Training can then be prescribed by identifying the load for each individual that causes a given decrement in velocity. This would be particularly useful in young athletes given the increased variability of sprinting kinematics and kinetics associated with maturation [17]. However, there is limited research using this approach, and to the authors’ knowledge, there has been very little research describing the responses of young athletes to resisted sprinting.

Using individual load–velocity profiles to prescribe training with a load that causes a given Vdec will provide practitioners with a simple method to standardize the training stimulus across individuals, with different training goals expressed relative to Vdec. The linear load–velocity relationship during resisted pulling leads to a parabolic power relationship. It has been demonstrated that a Vdec of 50% maximizes power output during sled pulling, and suggested athletes should train with loads that cause this reduction in velocity if the goal is to maximize power gains during sprinting [16]. The recommended loads, however, are far greater than any load ever studied in young athletes. The study also confirmed the linearity of the load–velocity relationship for a range of individuals (r^2^ > 0.97) and showed that there was large between-participant variation in the load that corresponded to a Vdec of 50% (69–96% BM). While these methods have been verified in adult athletes, it is unknown whether this would be the same for youth athletes given that they undergo anatomical, physiological, and biological variations due to the maturation process [18]. It is possible that the variability may exist to an even greater extent in resisted sled pulling as load increases in young athletes due to the differences in maturity, size, and strength [15].

While the load that optimizes power during sled pulling has been established, other optimization strategies may be needed to achieve different training goals. Extending on the work of Cross et al. [16], different percentages of Vdec may represent training zones for either more speed or force orientated training. Other researchers [7,8] have suggested limiting Vdec to <10% as the load to optimize the maintenance of kinematics while providing a resistive stimulus. More recently, it has been suggested that prescribing a Vdec <35% or >65% may target speed–strength and strength–speed qualities, respectively [13]. Theoretically, it is clear that Vdec can be used to prescribe different training intensities during resisted sprinting, but to date, no research has examined the ability of individual load–velocity profiles to identify optimal loads across a range of training zones in young athletes. The aims of the study are to examine the usefulness of individual load–velocity profiles and the amount of between-athlete variation associated with the Vdec approach to prescribe training loads during sled pulling in young athletes. The authors hypothesize that the Vdec approach is a reliable, effective, and precise way of prescribing sled load to young athletes.

## 2. Materials and Methods

### 2.1. Subjects

Seventy male high school team sport athletes from two sports, rugby and lacrosse (16.7 ± 0.9 years; height, 1.77 ± 6.9 cm; weight, 75.6 ± 10.9 kg; post-peak height velocity 1.8 ± 0.8 years and Vmax; 8.08 ± 0.49 m/s) were recruited to participate in this study. All subjects’ biological maturity was established as post-peak height velocity (PHV) using a non-invasive method with reliability within 0.5 years of calculating the age at PHV according to Mirwald et al. [19]. All subjects had a minimum of one-year resistance training experience and were healthy and injury free at the time of testing. Written consent was obtained from a parent/guardian and assent from each subject before participation. Experimental procedures were approved by the West Chester University institutional ethics committee. The study was conducted according to the Declaration of Helsinki.

### 2.2. Study Design

To determine the load–velocity relationship of un-resisted sprinting and sled pulling in youth athletes, seventy male subjects performed one un-resisted and three resisted sprints during a familiarization and the subsequent data collection session. A subset of participants (*n* = 15) was used to examine the reliability of sled pulling, repeating the protocol on three separate occasions separated by seven days. Resisted sprints were completed with a range of loads to allow the load–velocity relationship to be modelled. The maximum velocity attained (Vmax) during each sprint was measured via radar gun. Using Vmax individual load–velocity relationships were then established for each subject and used to identify loads that corresponded to a Vdec of 10, 25, 50, and 75%.

### 2.3. Procedures

All subjects reported one week prior to the first data collection, where they were familiarized with the equipment and testing procedures. Testing procedures were completed in dry conditions and on an outdoor 4G artificial turf field with sprint lanes set-up at a cross wind. A randomized counter balance design was implemented during data collection. Subjects were required to abstain from high-intensity training in the 24 h prior to the testing session. Subjects wore running shoes and comfortable clothing. A radar device (Model: Stalker ATS II, Applied Concepts, Dallas, TX, USA) was positioned 10 m directly behind the starting position and at a vertical height of 1 m to approximately align with the subject’s center of mass as per the recommendation of Simperingham et al. [20].

Subjects started from a standing split stance position and sprinted in a straight line for a recorded distance of 30 m with maximal effort for un-resisted efforts and 20 m for resisted efforts. A set of cones was placed 2 m in front of each 30 and 20 m markers to ensure maximal effort and achievement of maximal velocity during the sprint. Distances were estimated from pilot testing to ensure Vmax was achieved without inducing additional fatigue. In all sessions, subjects performed a standardized dynamic warm up consisting of sprint mechanics, dynamic stenches, and body weight exercises followed by two submaximal effort sprints (70% and 90% of self-determined maximal intensity) before completing maximal effort sprints. A minimum of four minutes and a maximum of six minutes of passive recovery was given between each sprint (un-resisted and resisted). Maximum velocity was gathered from the radar gun for all trials. Software provided by the radar device manufacturer (STATs software, Stalker ATS II Version 5.0.2.1, Applied Concepts Dallas, Dallas, TX, USA) was used to collect raw velocity data throughout each sprint.

#### 2.3.1. Un-Resisted Sprinting Protocol

Subjects were instructed to approach the start line and stand in a split stance with their preferred foot to jump off in front and kicking dominant foot behind. Subjects were instructed to sprint through a set of cones placed at 32 m.

#### 2.3.2. Resisted Sled-Pulling Protocol

**Figure 1:**
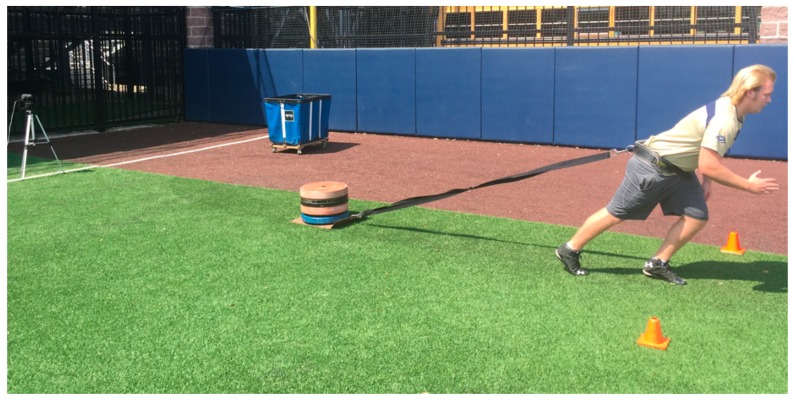
An example of the athletes starting stance and setup for resisted sled pulling.

Subjects received the same identical setup, instructions, and cues as per the un-resisted sprints. The heavy-duty custom-made pull sled (8.7 kg) was placed 3.3 m behind the subject attached to a waist harness by a non-elastic nylon tether. Subjects were instructed to take up all the slack in the tether to ensure no bouncing or jerking as they initiated the sprint. An example of this setup is illustrated in Figure 1. Participants were instructed to sprint through a set of cones placed at 22 m. The first resisted trial used an absolute load of 27 kg including the weight of the sled, participants then completed sprints with a minimum of three additional loads increasing in increments of 20% BM (+20, 40, and 60% BM). The load range was based on pilot testing, which determined the range of loads that reduced an athlete’s velocity by values above and below 50% of un-resisted Vmax and would allow individual load–velocity relationships to be calculated. Loads were selected to fall within the desired velocity decrement thresholds above and below 50% Vmax but not to induce unnecessary fatigue during maximal efforts.

### 2.4. Load–Velocity Relationship and Load Optimization

**Figure 2:**
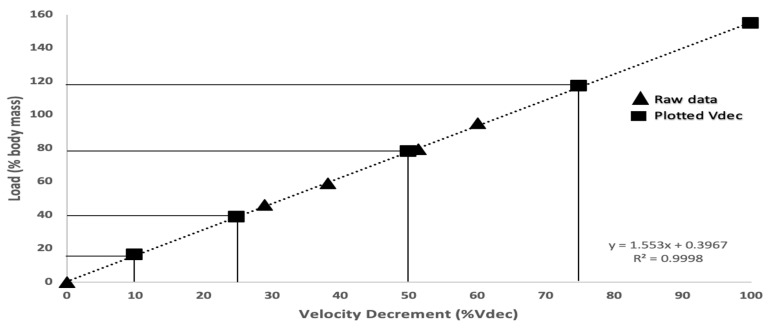
An example of the load–velocity relationship for one subject. The raw data (▲) shows the maximum velocity (Vmax) collected during resisted and un-resisted sprints. Using the linear relationship between load and velocity, the plotted Vdec (■) shows the calculated loads corresponding to a 10, 25, 50, 75, and 100% decrement in velocity.

Maximum sprint velocity was obtained for each un-resisted and resisted trial. The individual load–velocity (LV) relationship was established for each participant and checked for linearity. The linear regression of the load–velocity relationship was then used to establish the load that corresponded to a velocity decrement of 10% (L_10_), 25% (L_25_), 50% (L_50_), and 75% (L_75_), with the slope of the line explaining the relationship between load and velocity. An example of this is illustrated in Figure 2.

### 2.5. Statistical Analysis

Raw data was filtered through custom-made LabVIEW software to determine the maximum velocity of each participant during each sprint. Data were reported as means and standard deviation (SD) to represent the centrality and spread of the data. In the smaller subset of participants (*n* = 15), reliability of Vmax and Vdec were examined across the three different trials by calculating the change in the mean to examine systematic bias. Random variation was then investigated by establishing the relative reliability using an intra-class correlation coefficient (ICC) and absolute reliability using the coefficient of variation (CV). Between-day pairwise analysis of reliability was assessed using Hopkins’ online Excel spreadsheet [21]. Simperingham et al. [20] have suggested thresholds for establishing the reliability of sprints using a radar gun as a CV < 10% and ICC > 0.70. The load–velocity relationship of youth athletes was described using statistics from the larger sample of *n* = 70. The strength of linearity of the load–velocity relationship was established for each participant and a one-way repeated measures ANOVA with Bonferroni post-hoc test used to confirm whether differences in Vmax occurred with increased loading. The relationships between variables were determined using Pearson’s correlation coefficients. The alpha level was set as *p* < 0.05 with analysis performed in SPSS (Version 23.0). The mean Vdec across all participants at each load was calculated and between-subject variability calculated using 95% confidence intervals.

## 3. Results

The reliability of the variables of interest for the sled pull can be observed in Table 1. No consistent pattern of change in the mean was evident across Vmax, Vdec or the slope of the load–velocity relationship across the three trials. The coefficient of variation for Vmax was always <10%, while for the slope of the LV relationship and Lopt it was always <5%. The ICCs ranged from 0.60 to 0.92, with the lowest ICCs associated with Lopt and acceptable relative reliability for the slope of the LV relationship and Vmax. However, when Lopt was expressed in absolute load (kg), very high relative reliability (<0.90) was reported. Pairwise analysis indicated that both relative and absolute random variation were stable across trials.

### Load–Velocity Profiling Results

**Figure 3:**
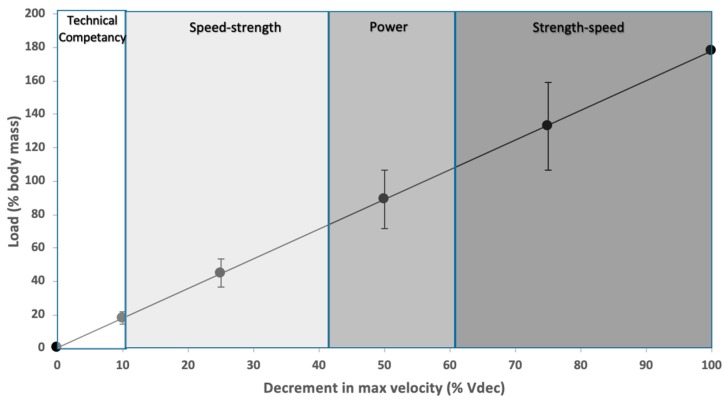
The linear mean load–velocity relationship for a group of *n* = 70 youth athletes with the loads that correspond to a decrement in velocity of 10, 25, 50, and 75 representing technical competency, speed–strength, power and strength–speed training zones.

In the large population of young athletes, the average Vmax achieved in un-resisted sprinting and with mean loads of 55 ± 3% BM, 75 ± 7% BM, 95 ± 10% BM, and 115 ± 14% BM were 8.1 m/s ± 0.59 s, 5.61 m/s ± 0.56 s, 4.47 m/s ± 0.54 s, and 3.74 m/s ± 0.47 s, respectively. Analysis revealed that Vmax at each load were significantly different to one another (*p* < 0.001). For all subjects, the load–velocity relationship was highly linear (*r* > 0.95), as was the case for the mean data across the group (*r* = 0.99). The mean load–velocity profile together with loads that correspond to a Vdec of 10, 25, 50, and 75% for a large group of youth athletes can be observed in Figure 3. Based on the individual load–velocity relationships, the Lopt that corresponded to a Vdec of 10, 25, 50. and 75% (95% CI) were 18 (14–21), 45 (36–53), 89 (71–107), and 133% (107–160) BM. Pearson’s correlation coefficients did not demonstrate a significant relationship between Lopt expressed as % BM and variables such as maturity, weight or Vmax.

## 4. Discussion

The purpose of this study was to examine the usefulness of load–velocity profiling and the between-athlete variation associated with load prescription during resisted sled pulling in young athletes. The highly linear nature of all individual load–velocity profiles confirms the validity of the approach. The study also established that optimized loads could be reliably identified for different decrements in velocity, suggesting the process can be used to consistently prescribe loads specific to a variety of training outcomes. Importantly, the study also highlights that there is relatively large between-subject variation in the loads that cause a given amount of Vdec. For example, the load that optimizes power, causing a Vdec of 50%, had a confidence interval spanning 71–107%. This individual variability is in agreement with previous research [16] and confirms that prescribing load simply as a given % BM for all individuals would be an invalid approach to prescribe training load in young athletes.

Reliability analysis demonstrated no systematic bias in any of the variables, suggesting the absence of any learning effects, which is in agreement with previous research in adult populations [5,9,16]. This is the first study to examine the reliability of resisted sled pulling in young athletes. When examining the CV across multiple loads for Vmax, it was found to demonstrate acceptable absolute reliability <10%. Optimizing load might be considered the variable of most interest for resisted sled training prescription, and this had low random variation with CVs < 5%. Intra-class correlation coefficients were acceptable (≥0.70) for nearly all Vmax comparisons. Although ICCs were lower for Lopt, when expressed in absolute loads they demonstrated very high levels (<0.90) of relative reliability. This finding reflects the more homogenous nature of Lopt when expressed relative to body mass versus the more heterogenous nature of Lopt when expressed as absolute load. The high reliability of the optimized loads for each training zone was due to the consistency of the load–velocity profile, with the slope of the individual relationships found to be reliable. Specific conditions of <10, 25, 50, and 75% of Vdec to correspond within zones of technical competency, speed–strength, power and strength–speed have been suggested in this study. However, based on the reliability of the load–velocity slope, researchers and practitioners could identify optimized loads that correspond to alternative target decrements in velocity. Specific Lopts could be reliably prescribed to young athlete’s dependent on the phase of the season such as heavier strength–speed zones during pre-season phases and lighter speed–strength zones closer to or within competition.

The high degree of reliability shown in the current study are congruent with previous research examining sled load prescription [5,16]. The lack of systematic bias and stable random variation across trials suggests there were no improvements in reliability across trials, which may be partly due to the familiarization to sled pulling prior to data collection. The results of the current study suggest that individual load–velocity profiles can be reliably used to identify optimized loads across a range of velocities. It is difficult to compare the data of the current study to previous research, due to the lack of research that has used sprint LV profiling in youth athletes. However, force–velocity and load–velocity profiling in other forms of resistance exercises in youth have been shown to be reliable (CV 0.7–6.8; ICC–0.94) [22]. The results of the current study suggest the method can be applied to youth athletes to provide an individualized approach to sled-load training prescription.

Resisted sprint training is a popular method of providing a sprint-specific resistive stimulus. Consequently, resisted sled pulling is a common training method examined by researchers [6,12,13]. However, little uniformity exists for sled-load training prescription. Unsurprisingly, the addition of greater load caused significant reductions in sprint velocity, allowing the load–velocity relationship to be modelled. The validity of the method is supported given the linear relationship between load and velocity; the current study demonstrated all individuals had a highly linear profile (*r* > 0.95) suggesting the approach can be applied to a large range of athletes. The loads corresponding to a Vdec of 10, 25, 50, and 75% were 18, 45, 89, and 133% BM, respectively. These loads are considerably higher than the majority of the literature previously examining sled pulling and far greater than loads considered heavy (20–30% BM) and very heavy (30+ % BM) in a review by Petrakos et al. [12]. Based on the current findings, loads of 20–30% BM would only be likely to cause modest decrements in velocity (<20%), and what are considered “heavy” loads may need to be reconsidered by both researchers and practitioners.

In agreement with recent research [16], there was a large amount of between-subject variation in Lopt for a given training outcome. Cross et al. [16] reported a range in load of 69–96% BM to cause a Vdec of 50% to optimize power. Similarly, the current study found that a Vdec of 50% resulted in loads ranging from 71–107% BM across a large group of youth athletes and this level of between-athlete variability was consistent across training zones. Although large variability was found between athletes, the Lopt expressed as % BM was not significantly related to weight, PHV or Vmax. Rumpf et al. [15] found significant differences on the effect of loading between pre- and post-PHV athletes; however, the current study found no significant relationship between levels of maturity and Lopt within a cohort of post-PHV athletes. Further research such as the assessment of strength and fat-free mass is needed to better explain the variability between athletes within a group of post-PHV. The findings of this study have major implications for sled-load training prescription for youth populations. While practitioners and previous research have traditionally prescribed loads based on % BM [15,23,24], this approach appears invalid. Based on the current findings, a given load prescribed as a set % BM could reduce the speed of one athlete by up to 50% more than that of another athlete. This would expose athletes to very different stimuli and would potentially lead to different chronic training adaptations. Prescribing training using individual load–velocity profiles provides a method to reliably target a given decrement in velocity within a desired zone of training such as technical competency, speed–strength, power and strength–speed. Furthermore, matching the training zone to the athlete’s force–velocity characteristics could potentially yield better training results than simply applying the same resistive load for all athletes [25]. However, further research is needed to better explain the between athlete variation and understand the chronic adaptations when undertaking this approach to sled-pull training in young athletes.

The majority of resisted sprint training research has primarily focused on the high-velocity end of the load–velocity relationship [7,8], ensuring minimal disruption to sprint mechanics by keeping velocity at >90% of the maximum. In the current paper, this has been termed the technical competency zone. This zone may be more applicable to sprinters who want to add a resistive stimulus while still achieving high velocities without affecting sprint mechanics closer to competition. With respect to maturation, technical competency zone training could be best utilized during pre-PHV in young athletes when technical acquisition of sprint mechanics is a priority due to the central nervous system development [26]. Alternatively, athletes of post-PHV who are undergoing increases in androgenic hormones and greater muscle cross-sectional area at the onset of puberty will benefit more with greater resistive loads to stimulate the ability to produce high amounts of horizontal force and impulse [10,15,27]. A recent review by Lesinski et al. [28] suggested that practitioners should emphasize higher intensities and force dominant capabilities of young athletes. Therefore, heavier resistive sled loads may be viewed as an extension of traditional resistance training, but applied horizontally rather than vertically. Recent research has begun to examine the use of heavier sled loads in adults [10,11,25], although apart from the current study only loads of up to 20% BM have previously been used with youth athletes [14,29]. More research is needed to understand chronic training adaptations to heavier sled loads, particularly when prescribed to cause a target decrement in velocity. 

## 5. Conclusions

In conclusion, the findings of the current study confirm our hypothesis that the load–velocity relationship is linear during sled pulling in young athletes. The slope and Vdec approach to sled-pulling load prescription were found to be reliable also. However, the load associated with a given Vdec varies across young athletes. The highly linear relationship between load and velocity and acceptable reliability of variables derived from individual load–velocity profiles allow for consistent sled-load training prescription in young athletes during a time in which development of speed is critical. The large variability in the amount of loading required to cause a target decrement in velocity further reinforces the need to adopt an individual approach to sled loading, particularly where the goal is to provide a consistent training stimulus across young athletes of varying size, strength, and training histories. Optimized loads for different training zones were reported in the current study and found to be reliable for technical, speed–strength, power and strength–speed zones. These zones can be used to help coaches periodize sled-loading parameters across a season, such as utilizing strength–speed zones during the off-season and speed–strength zones as competition approaches. Most importantly, the load–velocity relationship was found to be reliable, which means practitioners could reliably prescribe training for any given decrement in velocity. This would allow coaches to qualitatively prescribe individual sled loads and zones of training based on the force–velocity characteristics of the individual athlete. Given the maturational differences across young athletes, sled types and surface practitioners should determine individual load–velocity profiles for athletes in their training environments to better target the desired training adaptation.

## Figures and Tables

**Table 1:** The reliability of maximal velocity (Vmax), the load corresponding to given decrements in velocity (Lopt), and the slope of the load–velocity relationship during resisted sled pulling. Results are shown as mean ± SD and reliability statistics (95% CI). CV—coefficient of variation; ICC—intra-class correlation; Vmax—maximum velocity; Lopt—optimal load.

Reliability of Sprint Variables		Mean			Change in Mean (%)		CV (%)		ICC	
		Trial 1	Trial 2	Trial 3	Trial 1–2	Trial 2–3	Trial 1–2	Trial 2–3	Trial 1–2	Trial 2–3
Vmax (m/s)	Un-resisted	7.9 ± 0.5	8.0 ± 0.4	7.9 ± 0.5	1.0	−1.5	2.8	2.1	0.84	0.88
					(−1.1–3.1)	(−3.1–0.0)	(2.1–4.4)	(1.6–3.3)	(0.64–0.95)	(0.68–0.96)
	27 kg	6.1 ± 0.8	6.1 ± 0.8	6.1 ± 0.7	−0.6	−0.7	4.9	3.1	0.84	0.92
					(−4.1–3.0)	(−1.7–3.2)	(3.6–7.6)	(2.3–5.0)	(0.60–0.94)	(0.79–0.97)
	+20% BM	5.2 ± 0.5	5.2 ± 0.5	5.1 ± 0.6	−1.1	−1.0	3.4	4.2	0.91	0.87
					(−3.5–1.4)	(−4.1–2.3)	(2.5–5.2)	(3.1–6.7)	(0.75–0.97)	(0.66–0.95)
	+40% BM	4.4 ± 0.6	4.1 ± 0.4	4.4± 0.6	−5.7	6.2	7.1	6.7	0.72	0.77
					(−10.5–−0.7)	(1.2–11.5)	(5.2–11.3)	(4.9–10.5)	(0.36–0.89)	(0.45–0.91)
	+60% BM	3.7 ± 0.6	3.5 ± 0.5	3.8 ± 0.6	−7.1	8.0	8.6	9.0	0.69	0.73
					(−13.4–−0.4)	(0.7–15.8)	(6.1–14.6)	(6.4–14.8)	(0.24–0.90)	(0.34–0.90)
Lopt (% BM)	10% Vdec	17 ± 1	17 ± 1	17 ± 1	−1.5	0.1	3.2	3.3	0.71	0.65
					(−4.3–1.4)	(−2.8–3.0)	(2.3–5.6)	(2.3–5.6)	(0.26–0.91)	(0.15–0.88)
	25% Vdec	42 ± 4	43 ± 3	42 ± 2	1.2	−0.7	4.8	3.7	0.60	0.60
					(−3.0–5.6)	(−3.9–2.6)	(3.4–8.3)	(2.6–6.4)	(0.07–0.87)	(0.08–0.87)
	50% Vdec	84 ± 7	85 ± 5	85 ± 4	1.3	−0.6	4.6	3.5	0.63	0.64
					(−2.7–5.5)	(−3.7–2.5)	(3.3–8.0)	(2.5–6.1)	(0.12–0.88)	(0.13–0.88)
	75% Vdec	125 ± 11	128 ± 8	127 ± 5	1.4	−0.9	4.8	3.7	0.60	0.60
					(−2.8–5.8)	(−4.1–2.4)	(3.4–8.3)	(2.6–6.4)	(0.07–0.87)	(0.07–0.87)
	Slope Load–Velocity	−1.72 ± 0.15	−1.72 ± 0.08	−1.72 ± 0.06	−0.7	0.4	4.0	2.2	0.71	0.75
					(−4.1–2.9)	(−1.5–2.4)	(2.8–6.8)	(1.6–3.8)	(0.23–0.91)	(0.30–0.92)
